# Effects of two different decellularization routes on the mechanical properties of decellularized lungs

**DOI:** 10.1371/journal.pone.0178696

**Published:** 2017-06-01

**Authors:** Jessica Julioti Urbano, Renata Kelly da Palma, Flávia Mafra de Lima, Paula Fratini, Leticia Lopes Guimaraes, Juan J. Uriarte, Letícia Heineck Alvarenga, Maria Angelica Miglino, Rodolfo de Paula Vieira, Renato Araujo Prates, Daniel Navajas, Ramon Farrè, Luis Vicente Franco Oliveira

**Affiliations:** 1 Experimental Cardiorespiratory Physiology Laboratory, Master’s Degree and PhD Program in Rehabilitation Sciences, Nove de Julho University, Sao Paulo, Brazil; 2 Laboratory of Clinical and Experimental Immunology, Department of Medicine, Division of Nephrology, Federal University of São Paulo (UNIFESP), Sao Paulo, Brazil; 3 Department of Surgery, Faculty of the Veterinary Medicine and Zootecny, São Paulo University, São Paulo, Brazil; 4 Unitat Biofísica i Bioenginyeria, Facultat de Medicina, Universitat de Barcelona-IDIBAPS-IBEC-CIBER de Enfermedades Respiratorias, Barcelona, Spain; 5 Master's and Doctoral Program in Biophotonics Applied to Health Sciences, Nove de Julho University, Sao Paulo, Brazil; Medical University of South Carolina, UNITED STATES

## Abstract

Considering the limited number of available lung donors, lung bioengineering using whole lung scaffolds has been proposed as an alternative approach to obtain lungs suitable for transplantation. However, some decellularization protocols can cause alterations on the structure, composition, or mechanical properties of the lung extracellular matrix. Therefore, the aim of this study was to compare the acellular lung mechanical properties when using two different routes through the trachea and pulmonary artery for the decellularization process. This study was performed by using the lungs excised from 30 healthy male C57BL/6 mice, which were divided into 3 groups: tracheal decellularization (TDG), perfusion decellularization (PDG), and control groups (CG). Both decellularized groups were subjected to decellularization protocol with a solution of 1% sodium dodecyl sulfate. The behaviour of mechanical properties of the acellular lungs was measured after decellularization process. Static (Est) and dynamic (Edyn) elastances were obtained by the end-inspiratory occlusion method. TDG and PDG showed reduced Est and Edyn elastances after lung decellularization. Scanning electron microscopy showed no structural changes after lung decellularization of the TDG and PDG. In conclusion, was demonstrated that there is no significant difference in the behaviour of mechanical properties and extracellular matrix of the decellularized lungs by using two different routes through the trachea and pulmonary artery.

## Introduction

Several lung diseases result in irreversible structural lung damage, with lung transplantation as the only therapeutic indication when the progression of the disease is advanced [1]. Considering the limited number of lung donors [2], decellularized lung tissue has been shown to be a potential alternative in engineering whole lungs suitable for transplantation [3,4]. A scaffold suitable for use in whole lung tissue engineering must first be devoid of cells and cell components before reseeding [5,6]. However, some decellularization protocols have demonstrated that the absence of cells can affect the structure, composition, or mechanical properties of the lung extracellular matrix (ECM) [7,8], which can affect the recellularization process.

The agents for lung decellularization can be applied by using two different infusion routes: the pulmonary vasculature and the airway tree [9]. A previous study from our group demonstrated that decellularization process by both routes did not induce any significant differences in the micro-scale local stiffness of the decellularized lung [10]. However, no data are available on how the different routes affect the bulk mechanical properties (mainly elastance) of the whole acellular lung. The mechanical properties of the decellularized lung are important in lung bioengineering because the need of ventilating the organ during the recellularization process [11].

Therefore, the aim of the present study was to compare the lung static and dynamic elastances by using two different routes through the trachea and pulmonary artery in the decellularization process.

## Materials and methods

### Animals and lung extraction

This study was performed on lungs excised from thirty 7–8 week old (17–18 g) C57BL/6 male healthy mice, following experimental procedure approved by the Ethical Committee for Animal Research of the Universidade Nove de Julho (protocol number 0038/2011). Was carried out in strict accordance with the recommendations in the Guide for the Care and Use of Laboratory Animals of the National Institutes of Health and all surgery was performed under anesthesia, and all efforts were made to minimize suffering.

**Fig 1:**
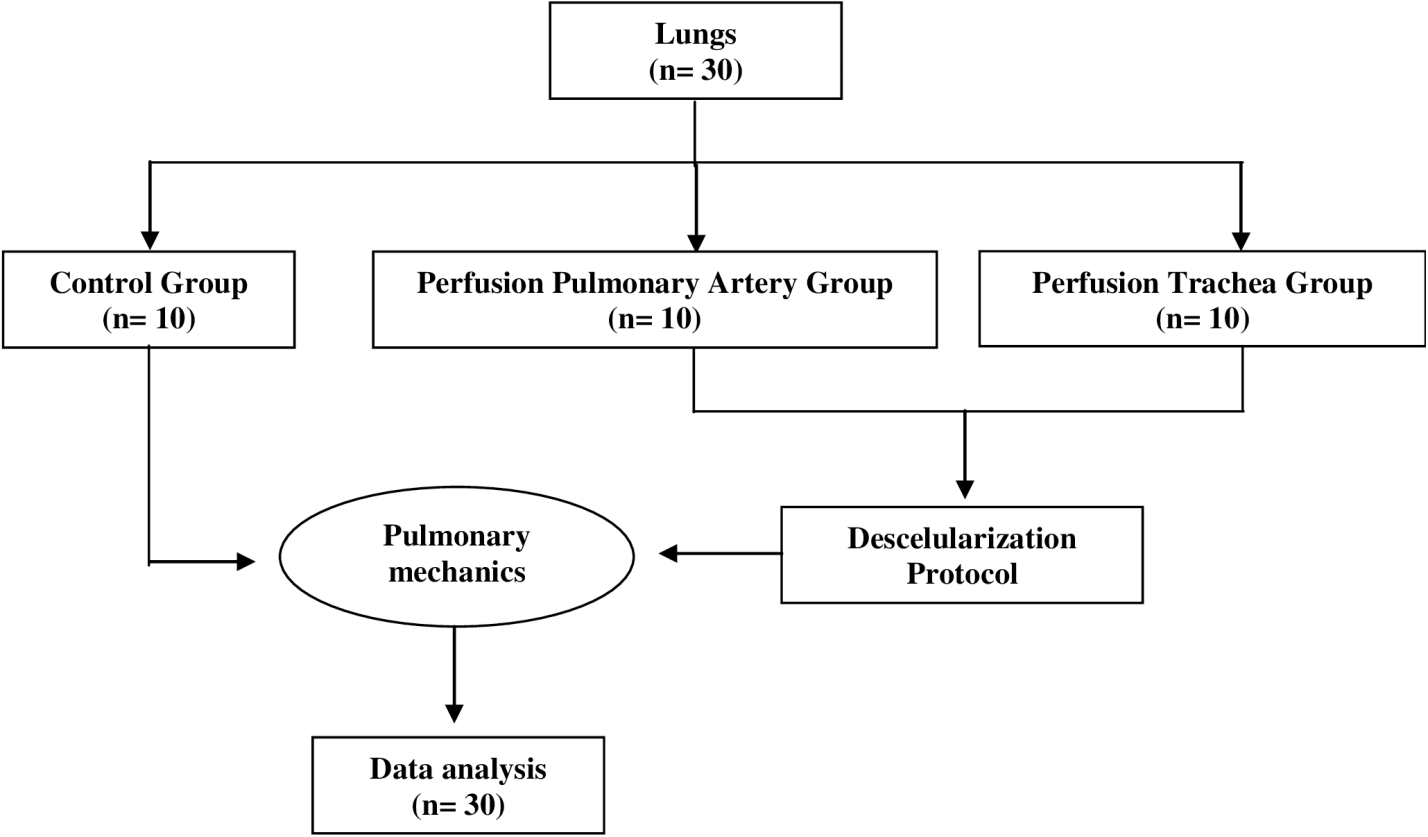
Flowchart of the study.

The animals were divided into three groups: tracheal decellularization (TDG, n = 10), pulmonary artery perfusion decellularization (PDG, n = 10), and control (CG, n = 10), as shown in Fig 1. The mice were anesthetized with intraperitoneal urethane (1 mg/kg), heparinized (250 U/kg), and sacrificed by exsanguination through the abdominal aorta. Immediately after euthanasia, the diaphragm was punctured, and the rib cage was cut to reveal the lungs. In the PDG, the pulmonary artery was cannulated, and the lungs were perfused with phosphate-buffered saline PBS containing 50 U/mL heparin (Sigma, St. Louis, Missouri, EUA) and 1 μg/mL sodium nitroprusside (SNP, Fluka, St. Louis, Missouri, EUA) via the right ventricle to prevent blood clot formation in the lungs. Finally, the heart, lungs, and trachea were dissected and removed en bloc, and stored in a −80°C freezer until the decellularization process was performed.

### Lung decellularization

The first step in the lung decellularization process involves thawing the lungs in a water bath at 37°C and freezing them again at −80°C; this cycle was repeated four times. After this first step, two different decellularization procedures followed, depending on whether the lungs were previously perfused or not.

The excised lungs without previous perfusion in the TDG were washed 6–8 times by tracheal instillation of 2 mL PBS containing streptomycin (90 mg/mL), penicillin (50 U/mL), and amphotericin B (25 mg/mL) until the liquid extracted from the lungs had a transparent appearance. This step was repeated with 2.5 mL de-ionized water several times, and subsequently treated with tracheal instillation of 2.5 mL 1% sodium dodecylsulfate (SDS) detergent. The lungs were subsequently kept in agitation for 24 h at room temperature in a 50-mL polystyrene conical tube with 20 mL of 1% SDS. The lungs were rinsed again with 2.5 mL PBS (with the antibiotic/antimycotic components described above) and maintained in 20 mL PBS in agitation for 24 h to finish the process for obtaining acellular lung scaffolds [12].

The PDG lungs, wherein the lungs were perfused before excision, had the trachea and pulmonary artery cannulated and placed into the experimental system; the trachea was cannulated and connected to a continuous positive airway pressure (CPAP) device that was set to provide a tracheal (i.e., transpulmonary) pressure of 10 cmH_2_O to inflate the lung at a physiological volume in an attempt to prevent atelectasis. The following decellularizing steps were followed through the pulmonary artery: 1) PBS 1× for 30 min, 2) deionized water for 15 min, 3) 1% SDS for 150 min, and 4) PBS for 30 min, at a pressure of 20 cm H_2_O [13,14].

### Assessment of lung elastance

We measured lung elastance to assess the potential changes induced by using both decellularization procedures on the mechanical properties of the whole lungs. Dynamic (E_dyn_) and static (E_st_) lung elastances were determined in the 30 lungs (10 CG, 10 TDG, and 10 PDG immediately after decellularization). To characterize the pressure–volume relationship under mechanical conditions similar to those in physiologically normal breathing, the lungs were subjected to conventional mechanical ventilation following a procedure described in detail elsewhere [12,15,16]. Briefly, the lungs were tracheally intubated, vertically suspended by gravity, and placed within a chamber (32°C and 100% humidity). A pneumotachograph was connected to the cannula inlet to measure tracheal flow by sensing the pressure drop across the pneumotachograph with a differential pressure transducer. Tracheal pressure was measured by connecting a pressure transducer on a side port placed between the pneumotachograph and cannula. The pneumotacograph inlet was then connected to the Y piece of a volumetric mechanical ventilator designed for artificial rodent ventilation [17]. The lungs were subjected to conventional ventilation with a quasi-sinusoidal flow pattern with 10-mL/kg tidal volume of mouse body weight, 100-breaths/min frequency, and 2-cm H_2_O positive end expiratory pressure, to counteract the absence of the physiological negative pleural pressure at rest. Flow and pressure signals from the transducers were analogically low-pass filtered, sampled, and stored for subsequent analysis.

The E_st_ and E_dyn_ were measured by using end-inspiratory airway occlusions achieved by pushing the corresponding control button of the mechanical ventilator. After an end-inspiratory occlusion, acellular lung pressure (DP1) rapidly decreased from the pre-occlusion value to inflection point (with pressure Pi), followed by a slow pressure decay (DP2) until a plateau pressure (Pel), corresponding to the elastic recoil pressure of the lung is reached. Whereas DP1 is associated with pressure dissipated against pulmonary resistance, DP2 reflects tissue viscoelastic properties or pendelluft. Taking into account the value of pre-inspiratory pressure (Po), lung Est was computed as the adjusted plateau pressure (Pel–Po) recorded after 5-s occlusion divided by the tidal volume. E_dyn_ was computed by dividing the adjusted inflection point pressure (Pi–Po) by the tidal volume [13,15,16]. For each native and decellularized lung, E_st_ and E_dyn_ were obtained as the means from five end-inspiratory occlusions, wherein each one was performed after 1-min normal mechanical ventilation.

### Scanning electron microscopy (SEM)

Slices of the decellularized lungs and control were prepared for imaging. The samples for SEM were fixed with 2% glutaraldehyde and 2.5% paraformaldehyde in 0.1-M cacodylate buffer (EMD Biosciences, USA) for 2 h at room temperature, rinsed in cacodylate buffer, and dehydrated through an ethanol gradient. The samples were further dehydrated in hexamethyldisilizane for 10 min and dried overnight, sputter-coated with gold, and analyzed by using the scanning electron microscope Hitachi Analytical Table Top Microscope TM3000 (Hitachi, Tokyo, Japan), with 15-kVa acceleration.

### Decellularization assessment

Three native and three decellularized lungs were fixed by submersion in 4% paraformaldehyde for at least 3 hours at room temperature, embedded in paraffin, and 5-μm sections mounted on glass slides. Following deparaffinization, in order to verify the absence of cellular DNA after the decellularization process, fluorescence was performed with the 4'-6-diamidino-2-phenylindole dye (DAPI). The images were captured by an EVOS FL fluorescence microscope and ensuring that the various areas of the sample will be cell free after decellularization.

In addition, the level of remaining DNA in the scaffold after using the perfusion procedure was assessed in three randomly selected decellularized lungs and in two native lungs. A sample of the right middle lobe of each lung was dried and weighted and its total genomic DNA was isolated using the spin-column based PureLink^®^ Genomic DNA Mini Kit (InvitrogenTM) according to manufacturer’s instructions. Double-stranded DNA yield was measured by spectrophotometry (NanoDrop 1000, Thermo Scientific) and normalized to sample tissue weight.

### Statistical analysis

After applied normality test (Kolmogorov–Smirnov test), homogeneity test of variance (average test Levene) was performed. Comparisons between the values obtained for Est and E_dyn_ measured in between each group were carried out by one-way analysis of variance and Tukey–Kramer test for multiple comparisons. Data are shown as mean±SE. The p value was considered statistically significant at a 5% level.

## Results

**Fig 2:**
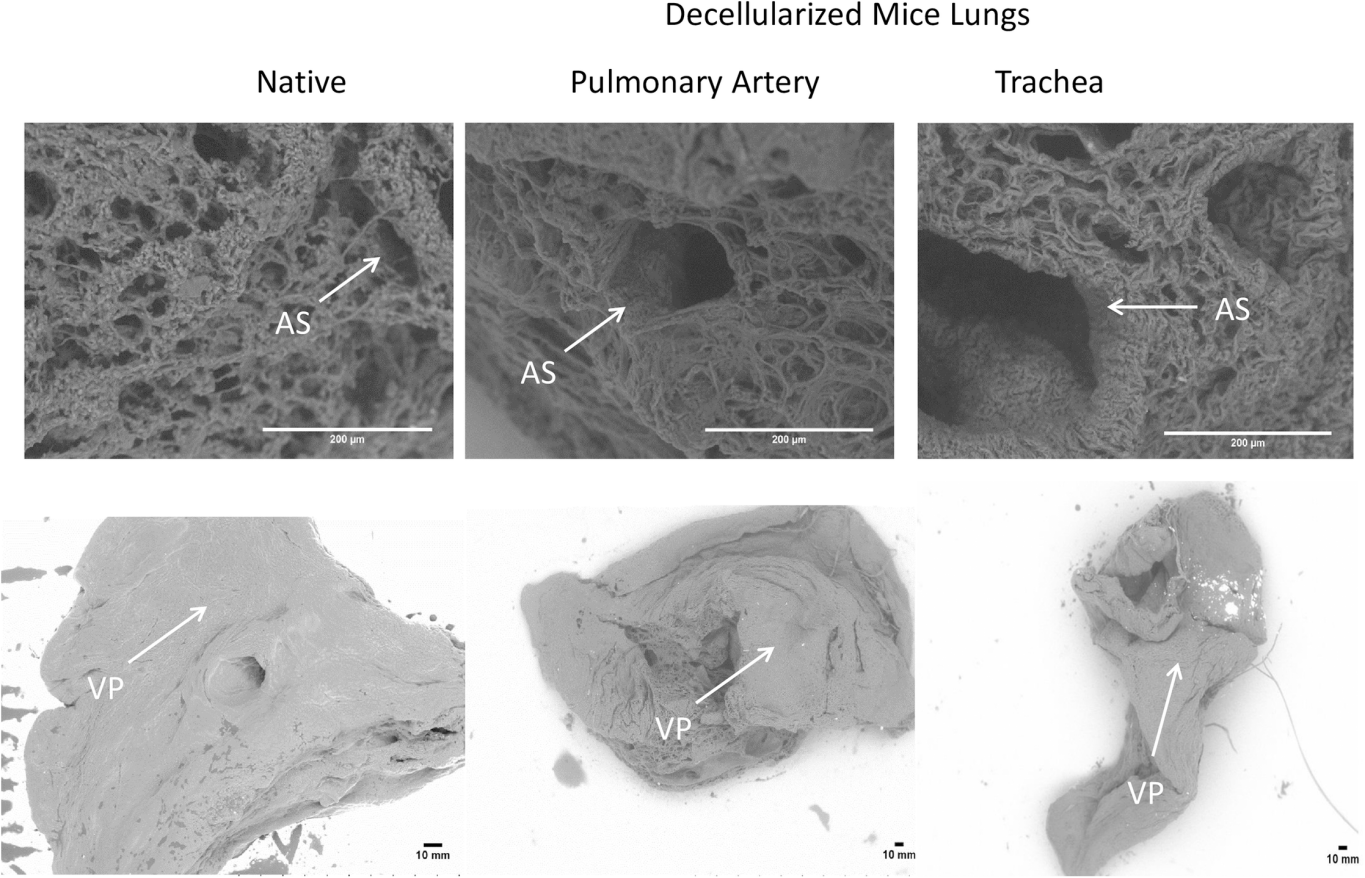
Representative examples of SEM images comparing sections of native and decellularized lungs (through pulmonar artery and trachea). AS: alveolar septum; VP: visceral pleura.

Scaffolds obtained from lung decellularization procedure (by using the pulmonary artery and trachea) compared with native lungs showed that the lung structures were relatively well maintained in all groups, as observed by SEM (Fig 2).

**Fig 3:**
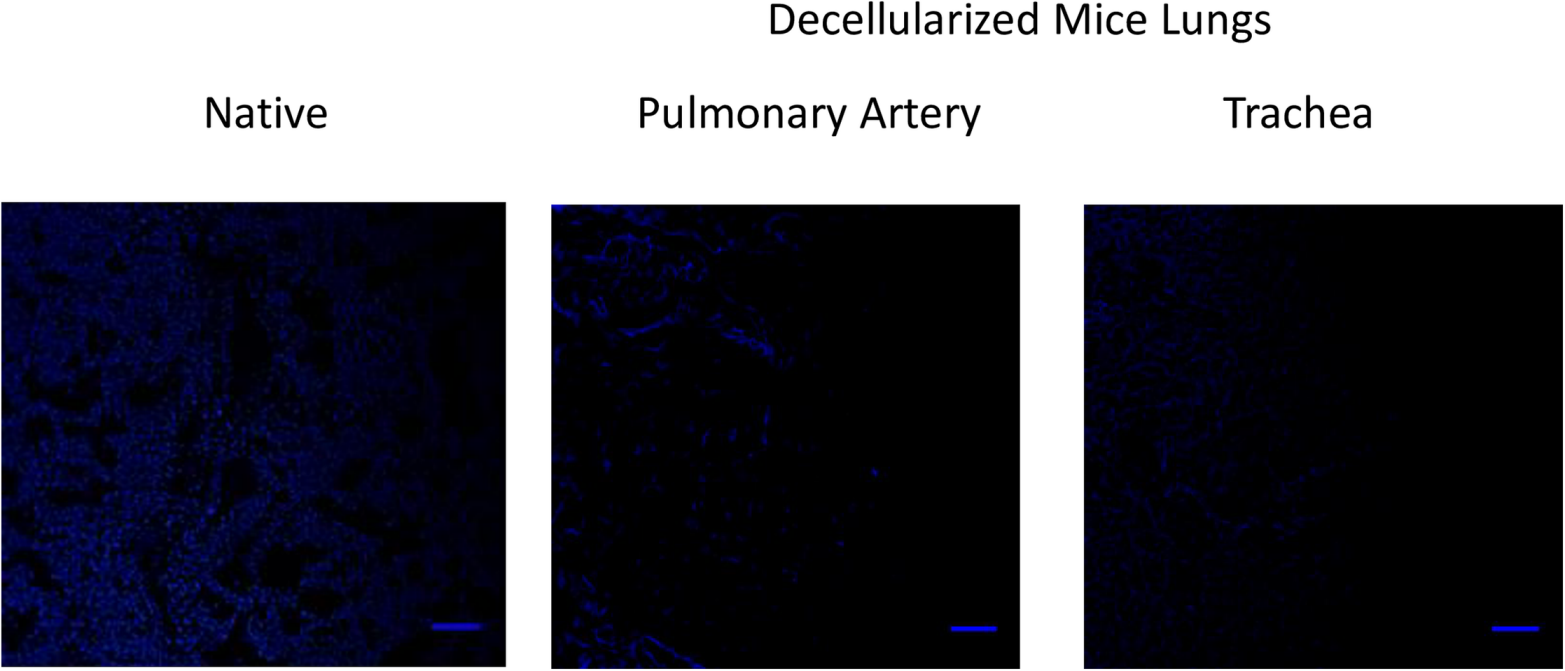
Representative image of DAPI staining in native and decellularized mouse lungs. Blue dots (absent in the decellularized lung) correspond to cell nuclei. Diffused blue staining in the acellular lung corresponds to autofluorescence of the extracellular matrix. Scale bar = 50 μm.

**Fig 4:**
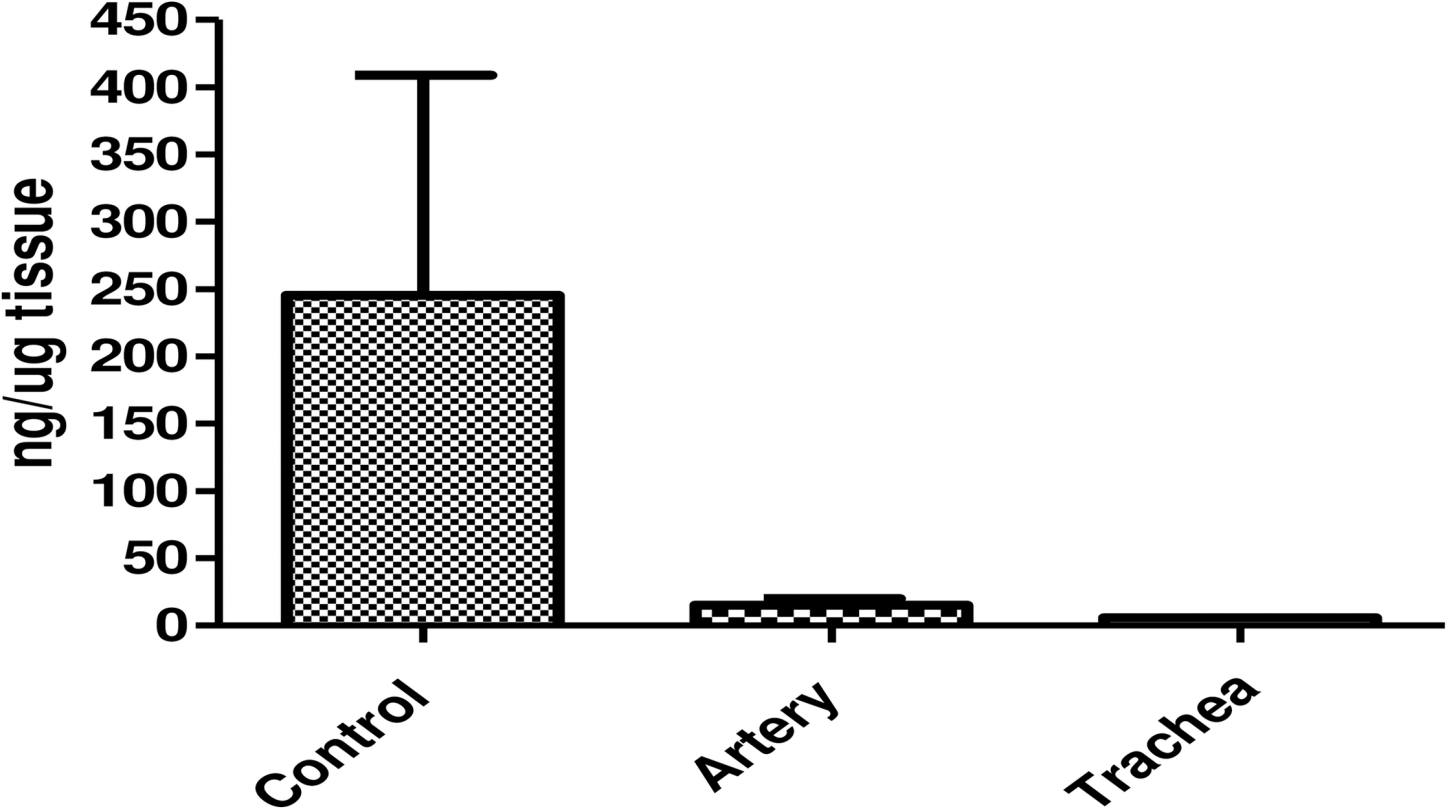
DNA content measured by spectrophotometry in native and decellularized lungs (through pulmonar artery and trachea).

In compared to native lungs, organ scaffolds lacked cellular nuclei assessed by DAPI (Fig 3) and genomic DNA content in the TDG was 5.8±2.13 ng/mg and PDG was 15.4± 4.6 (below the 50 ng/mg suggested by Crapo et al[18]) representing 1.7%(TDG) and 4.6% (PDG) of DNA content in the native lung (330 ng/mg) (Fig 4).

**Fig 5:**
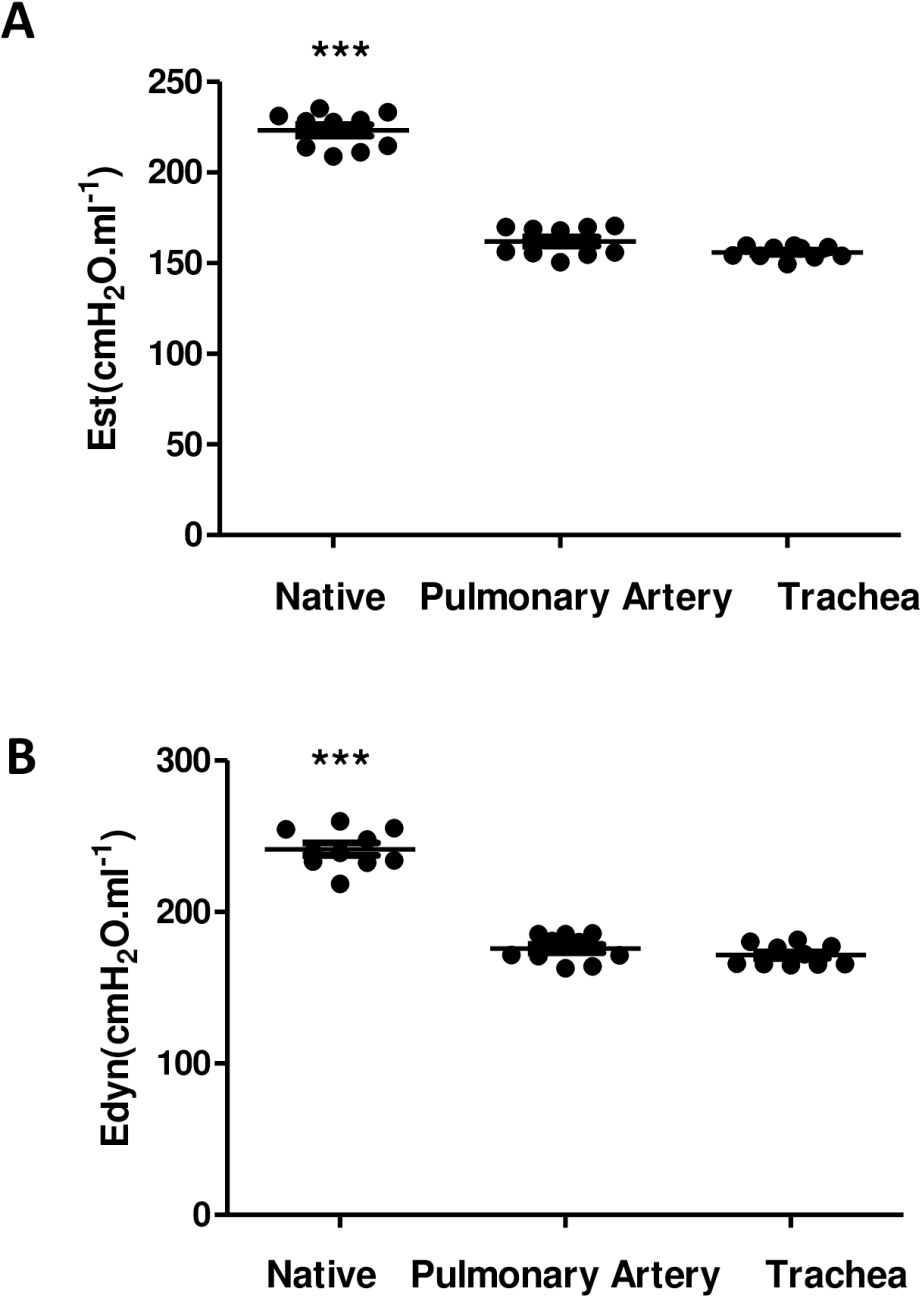
Lung mechanics. (A) Static (Est) and (B) dynamic (Edyn) elastances in native and decellularized lungs (through pulmonar artery and trachea) determined by the end-inspiratory airway occlusion method. Data are mean ± SE. ***: p < 0.01.

As shown in Fig 5, the lung elastance values (E_st_ and E_dyn_) measured in the acellular lungs were very close regardless decellularization was carried out through the pulmonary artery or the trachea (Eest = CG: 223.28±3.17; PDG: 162.0.4±2.53;TDG: 156.0.2±1.08, Edyn = CG: 241.47.±4.06; PDG: 175.84±2.78; TDG: 171.52±2.17), with these elastance values being lower than those corresponding to the native lung, determined by the end-inspiratory airway occlusion method.

## Discussion

Creating an acellular lung scaffold that is a suitable substrate for recellularization is known to be challenging. The mechanical properties and lung structure after decellularization processes are likely critical parameters when defining optimal decellularized scaffolds. Therefore, we demonstrate in the present study that applying both routes for lung decellularization, vascular and airway, resulted in a significant decrease in lung elastance, apparently maintaining pulmonary structures such as the alveolar septum and visceral pleura.

The detergent-based approach is one of the most frequently used among the methods for lung decellularization. In several studies from our laboratory, we perfused the SDS detergent through the trachea [12,15,16] and pulmonary artery [10,13,14], which resulted in an acellular lung with retention of specific ECM components and native cell population removal, which determines an optimal decellularization process. In this study we used the same protocol that was previously applied to lung decellularization with SDS through the trachea [12] and pulmonary artery [13]. Both protocols demonstrated preserved alveolar septum and visceral pleura, which are important pulmonary structures that determine optimum decellularized lungs, similar to the previous results. Wang et al., 2016 [9], recently compared the same routes for lung decellularization, the trachea and pulmonary artery, and demonstrated preserved ECM, but the airway structure and alveoli architecture of the pulmonary decellularized lung was partially destroyed. This probably occurred because of the high flow applied in the pulmonary artery during the decellularization process, unlike the constant physiological pressure applied in our protocol, which maintained the lung structures.

We used our experimental approach to measure the lung mechanical properties, which would provide novel data on the relationship between E_st_ and E_dyn_ elastances measured in the acellular lungs after end-inspiratory occlusion [12,13]. After both lung decellularization protocols, TDG and PDG, the viscoelastic system from the acellular lung was reduced compared with the native lungs, which was probably due to the elimination of lung cells (i.e., type II alveolar epithelial cells), which secrete lung surfactant thereby increasing lung compliance. Although not measured, it is expected that the changes we found in Est and Edyn would translate into the lung quasi-static relationship [7]. Therefore, considering that no damage was observed on the lung structures, this alteration in viscoelasticity will probably be restored during the repopulation process, and future research should be conducted in this regard.

In conclusion, we have demonstrated that no differences were found in the behavior of mechanical properties and structure damage of the decellularized lungs by using the trachea and pulmonary artery routes through to apply the decellularizing solutions. Therefore, this study provides information that could be relevant to produce a viable lung scaffolds for cell repopulation and future lung transplantation.

## Supporting information

S1 File Data mechanics. (XLS)
